# Treatment of grade 3B open tibia fracture by segmental resection and bone transport: A case report and literature review

**DOI:** 10.1016/j.ijscr.2024.110539

**Published:** 2024-10-30

**Authors:** G.T. Alpharian, Y.S. Robiady

**Affiliations:** a Advanced Trauma Division, Department of Orthopaedics and Traumatology, Hasan Sadikin General Hospital, Universitas Padjadjaran, Bandung, 40161, Indonesia; b Department of Orthopaedics and Traumatology, Hasan Sadikin General Hospital, Universitas Padjadjaran, Bandung, 40161, Indonesia

**Keywords:** Gustilo Type IIIB open tibia fracture, Segmental resection, Bone transport, Ilizarov technique, External locking compression plate

## Abstract

**Introduction and importance:**

Open tibial fractures represent the most prevalent type of open long bone fracture, constituting 13.7 % of all open fractures, typically resulting from road traffic accidents and falls from a standing position. The AO Trauma Foundation has developed comprehensive treatment procedures that encompass wound irrigation and debridement, fracture stabilization, and either delayed primary wound closure or early flap coverage. Managing Gustilo IIIB tibial fractures in adults poses problems due to elevated complication rates, increased infection risk, and prolonged union times. Consequently, multi-surgical intervention is necessary for the management of Gustilo Type IIIB open tibial fractures. Additional research on a related topic are compared to furnish a thorough summary of the existing knowledge concerning the effective care of Gustilo Type IIIB open tibial fractures accompanied by significant muscle rupture. Our objective is to assess the surgical efficacy of bone transport (Ilizarov and External LCP Technique) combined with segmental resection for the treatment of Grade IIIB open tibial fractures.

**Case presentation:**

A case study of a patient with a Gustilo Type IIIB tibial fracture featuring a 7 cm bone defect, managed through segmental resection and bone transport utilizing the Ilizarov technique. We assessed the patient periodically following each surgical procedure. We transition from Ilizarov to an external Locking Compression Plate (LCP) till the ultimate consolidation of distraction osteogenesis. The outcomes were assessed clinically and radiologically to evaluate the patient's leg function, infection status, and bone union.

**Clinical discussion:**

Open tibial fractures accompanied by bone and soft tissue defects pose significant challenges for achieving both fracture union and wound healing. External fixation is a commonly employed technique for the management of exposed tibial fractures. To address the deficiency, we performed segmental excision and bone translocation with the Ilizarov technique and external LCP. LCP serves as a less cumbersome and more tolerable external fixator compared to other external fixators. The sole worry surrounding the LCP external fixator was its sufficient stability for early weight-bearing.

**Conclusion:**

The Ilizarov method efficiently treats complicated fractures with significant bone and soft tissue abnormalities. Subsequently, we can employ external LCP to facilitate bone regeneration before doing bone grafting and internal fixation. Consequently enhancing patient comfort during routine activities.

## Highlights

• Complex fractures with extensive bone and soft tissue defects can be effectively treated using the Ilizarov method.
• The segmental fracture of bones, hence the bone loss of this case, and defect tissues around the site need to be removed and debrided with segmental resection.
• An external LCP can be employed to aid in bone regeneration.

## 1 Introduction

Open tibial fractures represent the most prevalent type of open long bone fracture, constituting 13.7 % of all open fractures. They are generally precipitated by vehicular collisions and falls from an upright position [1]. Open tibial fractures are intricate injuries characterized by diverse outcomes and prognoses, leading to prolonged hospitalization and an elevated Injury Severity Score [2]. The grading of open fracture severity significantly impacts therapeutic strategies and patient outcomes, so a classification system is widely employed during diagnosis [3].

Gustilo and Anderson categorize and assess the extent of soft tissue damage in open fractures, while also examining its impact on infection rates, with IIIB injuries defined by considerable soft tissue injury, periosteal stripping, and exposed bone [4]. Open tibial fractures require interdisciplinary management to get optimal outcomes. The AO Trauma Foundation has developed comprehensive therapeutic protocols [7]. Treatment alternatives encompass wound irrigation and debridement, fracture stabilization, and either delayed primary wound closure or early flap coverage [5].

Managing Gustilo IIIB tibial fractures in adults is challenging due to elevated complication rates, heightened infection risk, and prolonged union periods. Patient compliance must also be considered in the management strategy, since it involves time-consuming management with many surgeries required and an extended outpatient control timeframe to ultimately attain an acceptable outcome. The probability of infection is intricately associated with the extent of tissue damage, contamination, and vascular impairment. Infection rates for Gustilo IIIB might go as high as 52 % [6]. Furthermore, patient attributes including smoking status, immunodeficiency, and diabetes have been shown to elevate an individual's susceptibility to infection [7].

We present a case of an open tibial fracture Gustilo IIIB accompanied with an open fibula fracture. Gustilo IIIA endured several open reduction and external fixation surgeries and has successfully achieved fracture healing without infection, despite inadequate patient compliance with treatment. This case report has been documented in accordance with the SCARE standards [8].

## 2 Case report

**Fig. 1:**
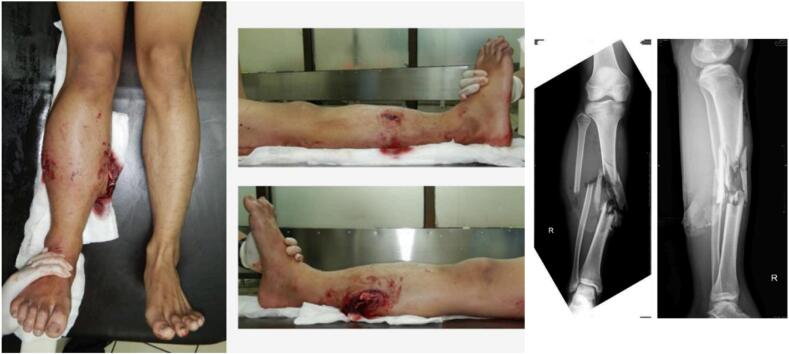
Degree of exposure of the fracture and soft tissue at the level of the right leg. (left and middle). Pre-surgical emergency radiographs (right).

A 19-year-old male patient presented to the Emergency Department at Hasan Sadikin General Hospital in Bandung with an open wound and deformity in the lower right leg following a motorcycle accident 1 h prior to admission. Patient consent was acquired. The initial assessment indicated normal airway, respiration, and circulation. No neurological impairment was observed. An open fracture was present in the right lower leg, accompanied by lacerations on both the lateral and medial aspects of the limb. Fig. 1.

A further examination of the right leg identified a lacerated wound on the lateral aspect of 3 × 3 × 1 cm, characterized by irregular edges, extending to the bone, with a possible partial rupture of the peroneus longus and brevis muscles, accompanied by deformity. A lacerated wound of 8 × 4 × 2 cm was observed on the medial aspect, characterized by irregular edges, including the bone, with suspected partial rupture of the soleus and tibialis posterior muscles, accompanied by deformity. Palpation indicated soreness, distal sensation was normal relative to the contralateral side, pulse of the posterior tibial artery was normal, and capillary refill time was under 2 s. The ankle's range of motion (ROM) was restricted due to discomfort. The patient was a non-smoker, with no history of medication or drug use, and no prior medical history.

Anteroposterior and lateral X-rays of the right lower leg demonstrated a complex multi-segmental fracture of the tibia and fibula with displacement, classified as type 42-C3. (Fig. 1) A 7 cm defect was present in the bone. The patient was diagnosed with a Gustillo Anderson type IIIB open fracture of the right lower leg. The fibula had a middle third oblique and displaced fracture, accompanied by partial rupture of the peroneus brevis, peroneus longus muscles, and the lateral head of the gastrocnemius. The tibial fracture was a comminuted fracture located in the middle third, accompanied by partial ruptures of the soleus, medial head of the gastrocnemius, flexor digitorum longus muscle, and tibialis posterior, as well as a complete rupture of the flexor digitorum longus.

We conducted several surgeries to manage this situation. An initial damage control surgery was conducted utilizing an external fixation with the Ilizarov device. At the six-month follow-up, we transitioned the Ilizarov device to an external LCP in response to patient complaints. The last procedure was conducted at the 18-month follow-up, involving the removal of the external LCP and conversion to internal fixation with bone grafting.

The patient received first treatment involving reduction and stabilization with external fixation. The patient was prepared and moved to the surgery room. The soft tissue deficit at the lateral aspect of the fracture site post-debridement was 3 × 3 × 1 cm, with verified partial ruptures of the peroneus longus and brevis muscles. The soft tissue deficit at the medial aspect of the fracture site post-initial debridement was 8 × 4 × 2 cm, with verified partial ruptures of the soleus, medial gastrocnemius, and tibialis posterior muscles.

**Fig. 2:**
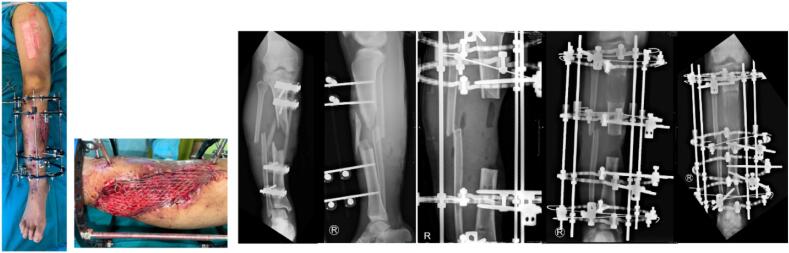
Intraoperative application of Ilizarov and application of split-thickness skin graft.

The fracture was realigned by using an external fixation device utilizing Ilizarov technique after the docking site was established and the fixed length was attained. The Ilizarov approach is regarded as the optimal therapeutic option since it facilitates bone defect reconstruction by distraction osteogenesis without the need for periosteal stripping. The surgery was conducted manually in the operating room with radiographs, with the position established through external fixation. The frame systems were stabilized following radiographic assessment. A split-thickness skin transplant was executed in this patient to rectify the deficiency at the fracture location (Fig. 2).

Notwithstanding the significant comminution at the fracture site, the reduction was deemed satisfactory, and the initial strategy was to employ external fixation as the definitive treatment option to prevent future compromise of the soft tissue envelope (Fig. 2).

**Fig. 3:**
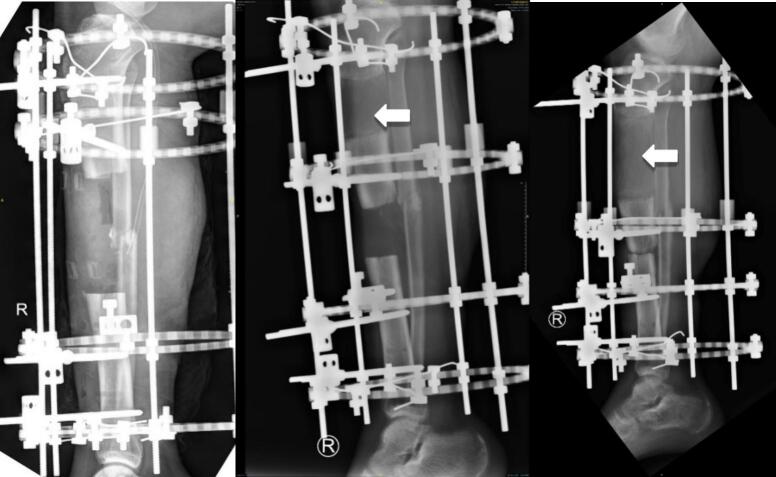
Radiograph images of the leg post Ilizarov application, 3 month follow up, and 6 month follow up, anterioposterior (AP) and right lateral (RL) projections of the right leg. White arrow indicates callus formation.

During the three-month follow-up, the bone transport segment remained in motion towards the docking point; hence, we opted to maintain the device. At the six-month follow-up, excellent integration was seen; the segment was already present at the docking site (Fig. 3). Nonetheless, the bacterial culture remained positive, indicating an elevated risk of infection should the fixation be altered to an internal fixation.

**Fig. 4:**
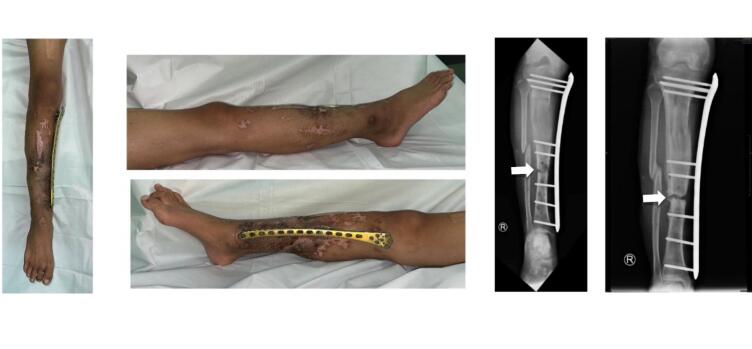
Images of the fracture and external fixation using locking compression plate. Radiograph images of the leg post LCP application (right).

The patient expressed discomfort with the Ilizarov device, which hindered daily activities. In light of the patient's condition and complaints, we opted to replace the Illizarov external fixation device with an external LCP. The proximal and distal primary bone pieces were externally stabilized using a locking device (Fig. 4).

The patient underwent additional clinical and radiographic evaluations at the 12-month follow-up, which indicated good bone development, absence of postoperative problems, and no symptoms of infection. An enhancement in the surgical and functional results was observed. The patient's soft tissues exhibited remarkable improvement, and callus development was evident (Fig. 4).

**Fig. 5:**
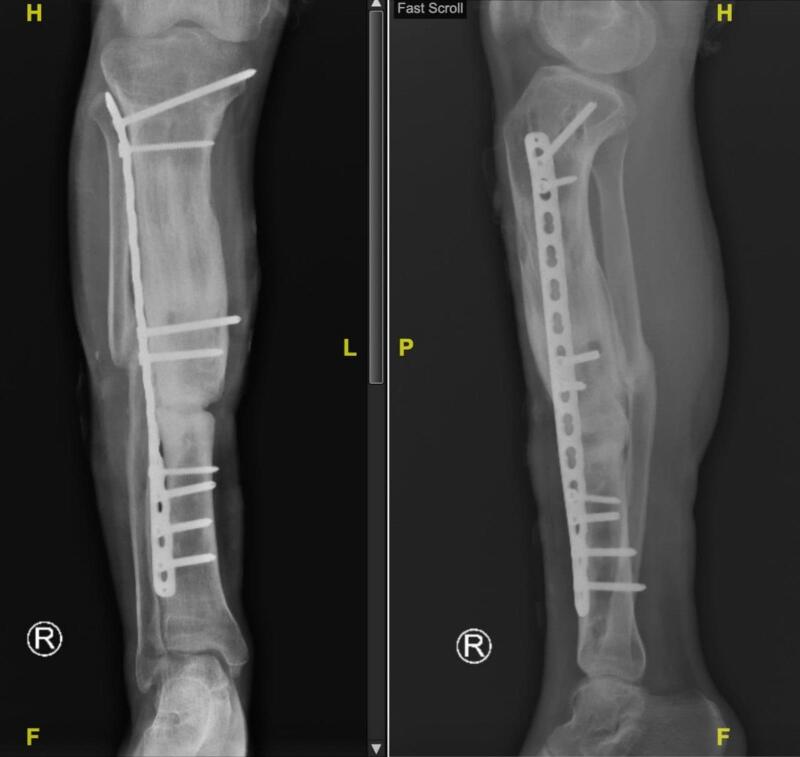
Radiograph images of the leg post ORIF + Bone Graft application in 21 months follow up, anterioposterior (AP) and right lateral (RL) projections of right leg. White arrow: callous formation.

**Fig. 6:**
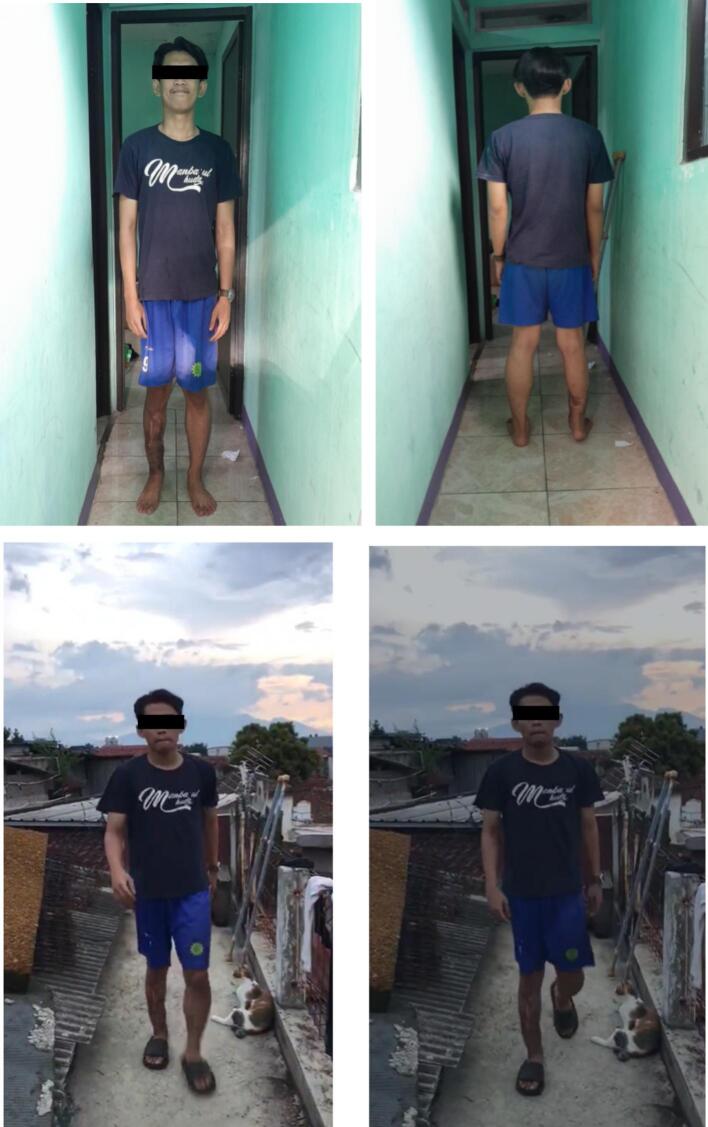
Clinical follow-up post-rehabilitation.

Ultimately, at the 18-month follow-up, the external LCP was excised and replaced with internal plating and bone grafting. Consistent follow-ups and meticulous x-ray surveillance facilitated the successful restoration of the tibial length. Three months post-surgery, acceptable bone development and function were attained (Fig. 5, Fig. 6).

The reason we prefer plating over nails is that the bone generation is already robust and healthy. If nails are used during the reaming process, there is a risk of damaging the bone generation, which will make the process more challenging. Ultimately, thermal necrosis will occur.

## 3 Post operative care, rehabilitation, outcome

Typically, as patients experience progressive enhancement in walking and balance, they may need only a single crutch prior to achieving independent mobility, contingent upon the patient's tolerance (Fig. 5). The gait, range of motion, strength, edema, discomfort, and surgical scars were all evaluated. Ice therapies, electrical stimulation, and heat were utilized to alleviate discomfort and swelling, facilitating expedited bone mending. Swimming and cycling facilitated muscle rehabilitation. Lifting exercises and leg lowering were conducted to facilitate the rehabilitation of the quadriceps femoris and gastrocnemius muscles. Patient adherence is crucial for optimal outcomes; nevertheless, in this instance, the patient frequently exhibited non-compliance.

## 4 Review of other studies

1. Mean bone healing time.

**Table:** 

Author, year	Number of Gustilo Type IIIB tibial fracture cases	Months from the first surgery to bone healing		
		Minimum	Maximum	Mean
Zhang Q et al. (2024) [9]	Total: 27			
Complete Union	27	13	133	28
Non-Union	–	–	–	–
Zhand D et al. (2020) [10]	Total: 4			
Complete Union	4	14	38	25
Non-Union	–	–	–	–
Barone N et al. (2022) [11]	Total: 1			
Complete Union	1	12	12	12
Non-Union	–	–	–	–
Pallaro J et al. (2015) [12]	Total: 7			
Complete Union	7	1,5	108	19
Non-Union	–	–	–	–
Papagiannis S et al. (2022) [13]	Total: 1			
Complete Union	1	24	24	24
Non-Union		–	–	–
Lerner A et al. (2023) [14]	Total: 1			
Complete Union	1	24	24	24
Non-Union	–	–	–	–
Pentela HK et al. (2023) [15]	Total: 1			
Complete Union	1	19	19	19
Non-Union	–	–	–	–
Saw KY et al. (2020) [16]	Total: 1			
Complete Union	1	15	15	15
Non-Union	–	–	–	–
Apivatthakakul T et al. (2007) [17]	Total: 1			
Complete Union	1	13	13	13
Non-Union	–	–	–	–

2. Treatment methods used.

**Table:** 

Author, year	Number of Gustilo Type IIIB tibial fracture cases	Methods
Zhang Q et al. (2024) [9]	Total: 27	Masquelet technique combined with free-flap technique (MFFT) and Ilizarov bone transport technique (IBTT)
Zhand D et al. (2020) [10]	Total: 4	Free tissue transfer and Ilizarov technique
Barone N et al. (2022) [11]	Total: 1	Debridement, IM nail exchange, cement spacer exchange and pedicled medial gastrocnemius, hemisoleus flap combined with split-thickness skin grafting (STSG) for coverage, and Ilizarov technique
Pallaro J et al. (2015) [12]	Total: 7	Debridement, femoral corticotomy, and Ilizarov technique with monoplane fixator
Papagiannis S et al. (2022) [13]	Total: 1	Debridement, reverse vascularized fasciocutaneous sural flap, STSG, external fixation, and Ilizarov technique
Lerner A et al. (2023) [14]	Total: 1	Ilizarov technique
Pentela HK et al. (2023) [15]	Total: 1	Ilizarov technique
Saw KY et al. (2020) [16]	Total: 1	Meshed split skin graft and Ilizarov technique
Apivatthakakul T et al. (2007) [17]	Total: 1	External fixation with broad locking compression plate, external fixation with LCP, free tissue transfer, corticotomy, distraction osteogenesis with Wagner lengthening device, and bone graft

Research indicated a total of 43 patients with Gustilo type IIIB tibial fractures.One to eight All patients in the eight investigations achieved bone union by the conclusion of their treatment regimen. The healing period required by patients varied from a minimum of 1.5 months to a high of 133 months. This statistics were influenced by the patients' comorbidities and adherence variables. Some patients were elderly, had bone cancer, and possessed other conditions that could impede their bone healing process or exacerbate their bone injury. None of the patients were diagnosed with an infection following the use of Ilizarov external fixation.

Multiple therapy modalities were administered to the patients in conjunction with the Ilizarov procedure. All patients underwent the Ilizarov bone transport technique in conjunction with treatments tailored to their specific problems. Tissue implants were performed in nearly all patients, with the procedure differing among trials. MFFT was conducted on 27 patients and yielded favorable outcomes. The other procedures were hemisoleus flap in conjunction with STSG, free tissue transfer, reverse vascularized fasciocutaneous sural flap, STSG, and meshed split skin transplant. A study by Apivatthakakul T et al. utilized the LCP and Wagner lengthening device as alternatives to the Ilizarov method. The outcomes were objectively acknowledged, and the patient regained normal ambulation following the healing of the bone. The radiographic evaluation revealed bone union 13 months post-admission. The study indicated that free tissue transfer was the employed strategy for addressing the patient's tissue defect issue. The various approaches employed in external fixation and distraction align with the primary focus of this work, which is consistent with earlier research including skin reconstruction, external fixation, and distraction.

## 5 Discussion

Open tibial fractures with osseous defects and significant soft tissue damage provide a difficulty for achieving both fracture union and wound healing. External fixation is a prevalent technique for managing exposed tibia fractures, noted for its low infection rates. It does not necessitate the positioning of devices at the fracture location and is distinguished by a reduced risk of vascular injury. It may serve as either a permanent or provisional fixing.

The Ilizarov technique of distraction osteogenesis can maintain fracture or nonunion alignment, promote osseous regeneration, minimize reliance on implanted devices, offer a robust foundation for soft tissue reconstruction, and facilitate full weight-bearing capacity.

Jitprapaikulsarn et al. formulated a treatment protocol for challenging Gustilo IIIB open fractures, incorporating internal fixation with plates and screws alongside soft tissue coverage utilizing a distally-based sural flap. The case presented segmental fractures and significant tissue anomalies that necessitated intervention. The segmental fracture of the bones needed to be excised initially to limit the possibility of impacting adjacent tissue, leading to bone loss in this instance. The compromised tissues surrounding the area required excision and debridement. Bone transfer and segmental resection were employed to excise segmental fractures and compromised tissues. After radical debridement, bone repair and soft tissue healing were achieved without any signs of infection.

For a 6 cm Ilizarov tibia lengthening, a duration of two months is required for distraction, followed by a minimum of four months of fixation with the extensive frame, and an additional two months of rehabilitation before achieving full weight-bearing and independent ambulation. A locking compression plate served as an external fixator that was less cumbersome and more readily accepted than earlier fixators. The sole concern regarding the LCP external fixator was its adequacy of stability for early weight-bearing. The documented example illustrates the management of a noncompliant patient necessitating many therapy interventions and modified schedules.

All treatments provided to the patients in the analyzed studies led to bone union and the alleviation of other comorbidities. All patients who underwent Ilizarov technique treatment successfully attained bone union, despite prior instances of fracture non-union. Pallaro J et al. discovered that the utilization of the Ilizarov process with a monoplane fixator facilitated expedited bone repair compared to the Ilizarov method used in isolation. This may impact the bone healing process; nevertheless, it is likely that additional factors, such as comorbidities, alter the duration of bone union. The efficacy of the monoplane fixator remains ambiguous due to the limited population size and the inclusion of individuals with comorbidities that may influence the bone healing process in the comparative samples.

We conducted early skin grafting to promote soft tissue healing before the split-thickness skin graft (STSG), as the wound bed was in ideal condition. The skin grafting technique utilized varied according on the patient's condition. Notwithstanding the variations in treatment methodologies employed, the patients achieved acceptable skin restoration. The patient had no signs of infection post-surgery when treated with medicines. While mesh split skin grafts exhibited the quickest time to attain bone union, their direct impact on the bone healing process remains questionable.

All patients who had both skin grafting and the Ilizarov technique ultimately attained bone union. The interval between the treatments varies; nonetheless, the combination therapy demonstrated that all patients achieved complete recovery after receiving both treatments within their treatment regimen. The efficacy of this case has been analyzed over a prolonged duration, and the patient can be handled proficiently. Nonetheless, a disadvantage of this method is the persistence of a docking site following treatment.

## 6 Conclusion

The Ilizarov approach, in conjunction with split-thickness skin grafting (STSG), can effectively address a complicated fracture accompanied by significant bone and soft tissue deficiencies. Initially, the segmental fracture of the bones, along with the associated bone loss and defective tissues surrounding the region, necessitates removal and debridement through segmental resection. Following radical debridement, bone regeneration and soft tissue healing occurred without indications of infection. Upon achieving the docking location, an external LCP may be utilized to facilitate bone regrowth.

External fixation is recognized as a prevalent technique owing to its diminished infection rates, elimination of direct manipulation at the fracture site, and less risk of vascular injury, providing both temporary and permanent fixation solutions. The adaptability of external fixators, permitting tailored configurations, improves stability for effective management. The Ilizarov method of distraction osteogenesis is effective in preserving reduction, promoting bone development, and offering a firm foundation for soft tissue restoration, facilitating complete weight-bearing.

The concurrent use of internal fixation and soft tissue covering in intricate open fractures exhibits efficacy. The case presented underscores the need for customized treatment strategies and modified timetables, particularly for patients with inadequate compliance, integrating diverse interventions to attain favorable results. This methodology can be applied to larger populations.

## Author contribution

Gibran Tristan Alpharian: Surgeon, Conceptualization, Visualization, Methodology, Writing and Supervision

Yandriyane Stephanie Robiady: Writing and Supervision.

## Consent

Written consent was obtained from the patient for publication of this case report and accompanying images. A copy of the written consent is available for review by the Editor-in-Chief of this journal on request.

## Ethical approval

Ethical approval for this study (LB.01.01/X.6.5/301/2024) was provided by the Ethical Committee of Medical Faculty, Padjadjaran University, Bandung, Indonesia on 2 August 2014.

## Guarantor

Gibran Tristan Alpharian.

## Research registration number

This is not a First on Man study.

## Funding

This research did not receive any specific grant from funding agencies in the public, commercial, or not-for-profit sectors.

## Conflict of interest statement

The authors declare that there is no conflict of interest regarding the publication of this paper.

## Data Availability

Not applicable.
